# Large age shifts in HIV-1 incidence patterns in KwaZulu-Natal, South Africa

**DOI:** 10.1073/pnas.2013164118

**Published:** 2021-07-09

**Authors:** Adam Akullian, Alain Vandormael, Joel C. Miller, Anna Bershteyn, Edward Wenger, Diego Cuadros, Dickman Gareta, Till Bärnighausen, Kobus Herbst, Frank Tanser

**Affiliations:** ^a^Institute for Disease Modeling, Bill and Melinda Gates Foundation, Seattle, WA 98109;; ^b^Department of Global Health, University of Washington, Seattle, WA, 98195;; ^c^Heidelberg Institute for Global Health, Heidelberg University, 69120 Heidelberg, Germany;; ^d^KwaZulu-Natal Research and Innovation Sequencing Platform, University of KwaZulu-Natal, Durban 4001, South Africa;; ^e^School of Engineering and Mathematical Sciences, La Trobe University, Melbourne, VIC 3086, Australia;; ^f^Department of Population Health, New York University Grossman School of Medicine, New York, NY 10016;; ^g^Department of Geography and Geographic Information Science, University of Cincinnati, Cincinnati, OH 45221;; ^h^Africa Health Research Institute, KwaZulu-Natal 4001, South Africa;; ^i^Medical Research Council/Wits-Agincourt Unit, University of the Witwatersrand, Johannesburg 2193, South Africa;; ^j^National Department of Science and Innovation, Medical Research Council, South African Population Research Infrastructure Network, Durban 4091, South Africa;; ^k^Lincoln International Institute for Rural Health, University of Lincoln, Lincoln LN6 7TS, UK;; ^l^School of Nursing and Public Health, University of KwaZulu-Natal, Durban 4041, South Africa;; ^m^Centre for the AIDS Programme of Research in South Africa, Durban 4013, South Africa

**Keywords:** HIV incidence, age distribution, antiretroviral therapy, HIV prevention

## Abstract

Recent declines in adult HIV-1 incidence have followed the large-scale expansion of antiretroviral therapy and primary HIV prevention across high-burden communities of sub-Saharan Africa. Mathematical modeling suggests that HIV risk will decline disproportionately in younger adult age-groups as interventions scale, concentrating new HIV infections in those >age 25 over time. Yet, no empirical data exist to support these projections. We conducted a population-based cohort study over a 16-y period (2004 to 2019), spanning the early scale-up of antiretroviral therapy and voluntary medical male circumcision, to estimate changes in the age distribution of HIV incidence in a hyperepidemic region of KwaZulu-Natal, South Africa, where adult HIV incidence has recently declined. Median age of HIV seroconversion increased by 5.5 y in men and 3.0 y in women, and the age of peak HIV incidence increased by 5.0 y in men and 2.0 y in women. Incidence declined disproportionately among young men (64% in men 15 to 19, 68% in men 20 to 24, and 46% in men 25 to 29) and young women (44% in women 15 to 19, 24% in women 20 to 24) comparing periods pre- versus post-universal test and treat. Incidence was stable (<20% change) in women aged 30 to 39 and men aged 30 to 34. Age shifts in incidence occurred after 2012 and were observed earlier in men than in women. These results provide direct epidemiological evidence of the changing demographics of HIV risk in sub-Saharan Africa in the era of large-scale treatment and prevention. More attention is needed to address lagging incidence decline among older individuals.

## Significance

HIV incidence has recently been in decline across some of the most intense epidemics in sub-Saharan Africa due to the scale-up of prevention and transmission-blocking treatments. Understanding whether declines in incidence are being felt equally across age and gender can help prioritize demographic groups where more effort is needed to lower transmission. We found that HIV incidence has declined disproportionately in the youngest men and women in a population with the highest HIV prevalence in the world. Shifts in the age distribution of risk may be the consequence of aging prevalence, prioritized prevention to younger individuals, and delays in age at infection from reduced overall force of infection. Our results highlight the need to expand age targets for HIV prevention.

Despite remarkable progress in the expansion and scale-up of HIV treatment and prevention, 1.7 million new HIV infections and 770,000 deaths from AIDS-related illnesses occur every year globally (1). Almost half of new HIV infections and 40% of AIDS-related deaths are in East and southern Africa. South Africa, with an adult HIV prevalence of 20%, has the largest HIV epidemic in the world, with 7.7 million people currently living with HIV (1).

Recent declines in adult HIV-1 incidence across high-burden communities of sub-Saharan Africa (SSA) are testament to the success of large-scale combination HIV prevention, including the expansion of antiretroviral therapy (ART) under universal test and treat (UTT) and the scale-up of voluntary medical male circumcision (VMMC) (2–5). When implemented at scale, large increases in viral load suppression from ART expansion are associated with substantial declines in population-level HIV incidence (2, 3, 5). The results from four large cluster randomized trials of UTT effectiveness demonstrated the feasibility of achieving high population ART coverage and viral load suppression through community mobilization (6–9). Yet, the results also showed the challenge of reducing population-level incidence with treatment alone (10–12).

At the same time that HIV incidence has fallen, the burden of HIV infection (HIV prevalence) has more than doubled in those >age 25 (13–17), a result of extended life expectancy among people living with HIV (PLHIV) on suppressive ART (18, 19). Mathematical models suggest that new HIV infections will become increasingly concentrated in older adults over time, a result of prioritized HIV prevention in youth (20), increasing HIV prevalence in older adults, and delayed age at infection with declining risk (12), yet no empirical evidence exists to support these projections.

Young age is one of the strongest predictors of HIV risk in SSA, a result of a sexual network structure that places younger individuals at greater risk of infection from older individuals (21–23), heterogeneous behavior and biology that increases exposure to and acquisition of HIV in younger women (24–28), and lower treatment coverage in younger PLHIV (29, 30). Disparities in HIV risk have supported efforts to target HIV prevention to younger individuals, especially to women <age 25 (23). As the epidemic ages, however, older cohorts of HIV-negative individuals now face a greater probability of exposure to sexual partners living with HIV than they did a decade ago (31), albeit with a reduced viral load because of ART scale-up (13–17, 32, 33). The expansion of treatment and primary prevention, by lowering the overall force of infection (transmission rate), may also increase the average time to infection, a dynamic observed in other infectious diseases (34–37). The fewer individuals who become infected earlier in life means those individuals stay in the population at risk for longer, thereby shifting the relative share of infections to older individuals. Age-structured sexual contact patterns specific to sexually transmitted infections can further magnify age shifts in risk, as individuals tend to mix preferentially with partners of a similar age-group (21). Older cohorts may thus account for a growing proportion of HIV incidence as the overall transmission rate declines in the era of large-scale treatment and prevention.

Despite enormous progress in efforts to scale-up treatment and prevention, HIV incidence remains well above epidemic control thresholds in high-burden communities (2–5, 38). More will be needed to identify subgroups with elevated risk to guide regional targets for HIV prevention over the next decade. The remarkable demographic and geographic heterogeneity characteristic of HIV epidemics across high-burden regions (39, 40) has ushered in an era of prioritizing the highest risk groups and geographies to achieve maximum population-level effect (41, 42). Understanding how the demographic landscape of HIV risk has shifted with the scale-up of combination HIV prevention is needed to realign prevention targets with current and future distributions of risk.

Here, we measure nonlinear age- and sex-specific trends in HIV-1 incidence between 2004 and 2019 from one of the world’s largest ongoing population-based cohorts in rural KwaZulu-Natal, South Africa, a region with among the highest HIV incidence rates in the world. Following recent work showing significant declines in adult HIV incidence in the region (2), we use statistical approaches that can flexibly reveal a wide range of HIV incidence patters to test the hypothesis that incidence has declined differentially by age and gender over a period spanning the expansion and scale-up of ART and VMMC. We provide plausible epidemiological explanations for the observed changes in the age distribution of risk. Our results have major implications for expanding demographic targets for HIV prevention in the era of UTT.

## Methods

### Study Setting.

The Africa Health Research Institute (AHRI) surveillance area is in the Umkhanyakude district of the northern KwaZulu-Natal province, with a population of ∼100,000 residents and ∼20,000 households (43–45). The region has among the highest adult HIV prevalence in the world, which has increased over time from 22% in 2005 to 37% in 2016 (46, 47). Adult (men 15–54 and women 15–49) HIV incidence is also among the highest in the world (2.3 per 100 person/years (py) in 2017) and has started to decline only recently (2). ART coverage among PLHIV increased steadily between 2005 to 2017 in men and women, though coverage remains low relative to the Joint United Nations Program on HIV/AIDS (UNAIDS) 90–90–90 targets (38.4% among men and 50.6% among women in 2017) (2). UTT was instituted in September of 2016, when ART became available regardless of CD4+ count (7).

### Data Collection.

Annual population-based HIV testing is nested within AHRI’s demographic surveillance system. Since 2003, trained fieldworkers have offered household HIV testing to all eligible participants (aged 15 to 49 y old among women and 15 to 54 y old among men), with eligibility expanding to participants 15 y of age and older after 2007. Dried blood spots are prepared from whole blood for HIV testing according to the UNAIDS’s and World Health Organization’s Guidelines for Using HIV Testing Technologies in Surveillance (48). HIV serostatus was determined by antibody testing with a broad-based HIV-1/HIV-2 enzyme-linked immunosorbent assay (ELISA) followed by a confirmatory ELISA (44).

Demographic and general health data are collected annually during household visits to record information on in and out migration, occupation, marital status, and sexual health, including number of lifetime sex partners and age at sexual debut. Self-reported circumcision status has been collected since 2009, when the local VMMC program started.

### Incidence Measurement.

HIV incidence was measured by directly observed changes in HIV serostatus among study participants (men aged 15 to 54 y old and women aged 15 to 49 y old) who tested HIV negative followed by at least one subsequent valid HIV test result. This method represents the gold standard for directly estimating incidence in a population cohort. Due to periodic testing, we did not observe the exact date of seroconversion for participants with an HIV-positive test result. We randomly imputed a seroconversion date between the last negative and first positive test dates under a constant hazard assumption (49). We then right-censored person time at the imputed seroconversion date or at the latest HIV-negative test date if the repeat tester did not seroconvert during the observation period. To account for the uncertainty of the single random-point method, we repeated this procedure to generate 300 imputed datasets for model fitting.

### Statistical Analysis.

Person-time incidence rates and 95% CIs were estimated by continuous age and year for men and women separately from fitted Poisson generalized additive models (GAMs) with a nonlinear interaction between study year (2004 to 2019) and age using penalized thin-plate regression splines (50) (*SI Appendix*). The models were estimated for each of 300 imputed datasets and point estimates and standard errors were combined using Rubin’s rules (51). The fitted models were used to estimate the smoothed age distribution of incidence per 100 py by sex and year. The age of peak HIV incidence was calculated as the age of maximum incidence for each year from the fitted model. The area under the curve was used to estimate the proportion of the incidence age distribution <age 25 by year. This metric estimates the proportion of new infections in individuals under 25 that would arise in a population at risk with a constant age distribution. Circumcision prevalence by continuous age and calendar year (2009 to 2019) was estimated among HIV-negative men included in the longitudinal cohort using smoothed GAMs with a logistic link and a nonlinear interaction term between age and year with smoothing using penalized thin-plate regression splines.

Poisson generalized linear models (GLMs) were used to estimate incidence rate ratios (IRR) and 95% CIs by categorical age-groups (15 to 19, 20 to 24, 25 to 29, 30 to 34, and 35 to 39, 40+) and categorical year corresponding to dates before and after UTT went into effect in 2016. Models for men were additionally run, adjusting for individual-level circumcision status to assess age shifts beyond those explained by differential VMMC uptake by age over time. To ensure that the results were robust to incomplete participation in the HIV testing of the underlying study population, Poisson GLMs were additionally run including inverse probability weights (IPWs) to account for missingness at random following methods used in previous cohort studies of HIV incidence (2, 3). IPWs were estimated from a logistic regression model with a probability of nonparticipation regressed on the following covariates: continuous age, number of migration events outside the study area that resulted in a temporary change of residence, length of time spent outside the study area during migration events, and HIV prevalence in the surrounding community by year.

### Ethical Approval.

All participants provided written informed consent prior to the household-based interview and collection of dried blood spots for HIV testing. The biomedical and ethics committee (BREC) of the University of KwaZulu-Natal, Durban, South Africa, provided ethics approval for data collection and use (BREC approval number BE290/16).

## Results

The longitudinal HIV incidence cohort included 22,406 individuals who tested HIV negative followed by at least one other test between 2004 to 2019. The cohort was young (27.4% aged 15 to 19 y old and 33.3% aged 20 to 24 y old), mobile (60.3% had ever spent time away from the study area and 84% of moves out of the study area were followed by a subsequent return), and experienced high levels of unemployment (90.5% ever experienced unemployment) (Table 1). The self-reported circumcision prevalence among men (ever circumcised) was 37.1% by the end of the study, increased over time between 2009 to 2019, and was higher among younger men (64.7% in men 20 y of age and 38.5% among men 35 y of age in 2019) (*SI Appendix*). ART initiation (ever-initiated ART) among PLHIV in the incidence cohort was low (33.2%) based on linked clinical records. The age at first marriage was 26.6 y, and 77.2% reported having had initiated sex; the average age of sexual debut (among those initiating first sex) was 17.2 y, and the mean number of reported lifetime sexual partners (among those who reported any lifetime partners) was 2.7. A summary of demographic characteristics comparing those included versus not included in the incidence cohort can be found in *SI Appendix*.

**Table 1.** Summary of demographic, behavioral, and HIV-specific characteristics at last study visit among individuals included in the population-based incidence cohort (HIV-negative test followed by at least one test)

	*N* = 22,406
	*n* (%)/mean (sd)
Female	12,605 (56.3)
Age (years)*	26.36 (9.4)
Age category (%)*	
15 to 19	6,141 (27.4)
20 to 24	7,465 (33.3)
25 to 29	3,266 (14.6)
30 to 34	1,377 (6.1)
35 to 39	1,029 (4.6)
40 to 54	3,128 (14.0)
Ever migrated out of the study area	13,502 (60.3)
Ever experienced unemployment^†^	18,750 (90.5)
Circumcised (ever among men)	3,039 (37.1)
Age at first marriage	26.63 (6.75)
Ever had sex	15,092 (77.2)
Age at first sex (years)	17.23 (2.4)
Lifetime partners (among ever had sex)	2.68 (3.2)
ART uptake (ever on ART among HIV+)	1186 (33.2)

All percentages are reported out of nonmissing responses.

* Age is calculated as the mean over follow-up.

^†^ Unemployment data unavailable for 2019.

**Fig. 1.**
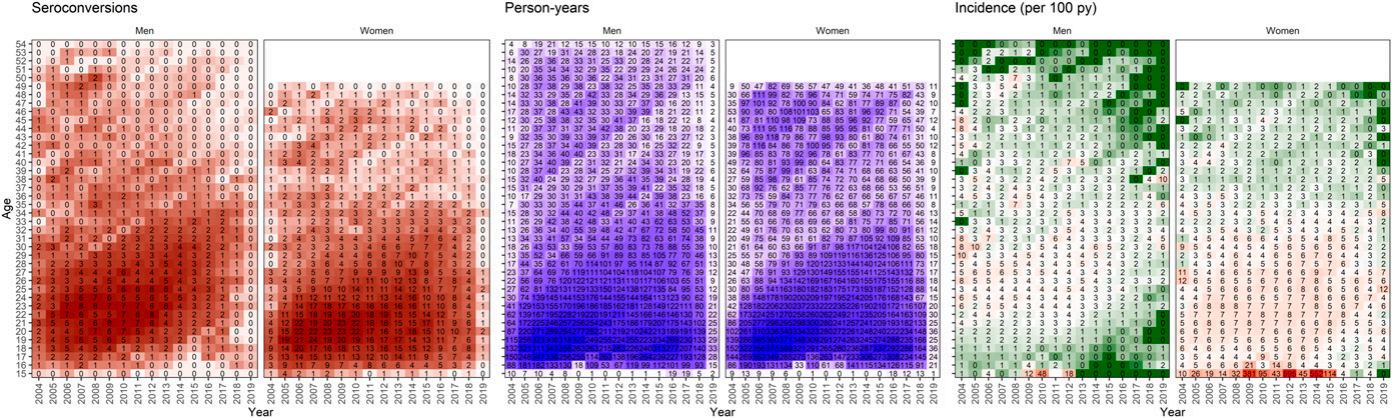
Crude number of HIV seroconversions, py, and incidence rates observed by continuous age and year stratified on sex and averaged across 300 datasets with seroconversion randomly imputed between the last negative and first positive. The numbers are rounded to the zero-decimal place for py and seroconversions. Incidence rates over 100 per 100 py were observed in age-year bins where at least one seroconversion occurred in less than 1 py.

Between 2004 and 2019, 3,574 new HIV infections were observed (887 among men and 2,687 among women) in 103,775 py of follow-up (43,629 py among men and 60,146 py among women) for an overall incidence rate of 3.4 per 100 py (2.0 per 100 py in men aged 15 to 54 y old and 4.5 per 100 py in women aged 15 to 49 y old). The median (interquartile range, IQR) time between last negative and first positive test among those who seroconverted was 3.0 y (IQR = 1.4 to 5.8 y) (*SI Appendix*). The py of follow-up and number of HIV seroconversions were concentrated in men and women <age 25, reflecting the young average age of the cohort (Fig. 1).

**Fig. 2.**
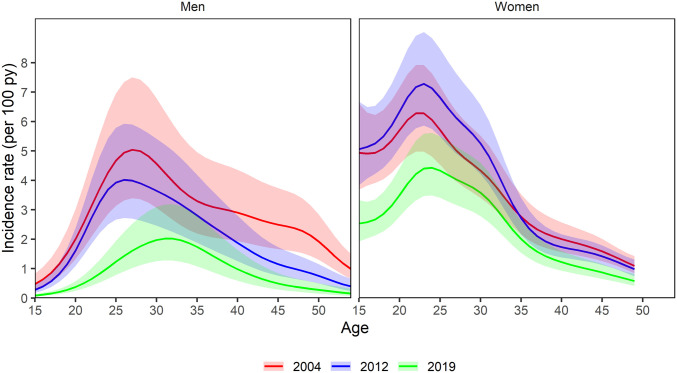
Year-specific cross-sections of incidence age distributions and 95% CIs from years 2004, 2012, and 2019. The largest difference in age distribution occurred in the latter half of the study period, between 2012 and 2019.

**Fig. 3.**
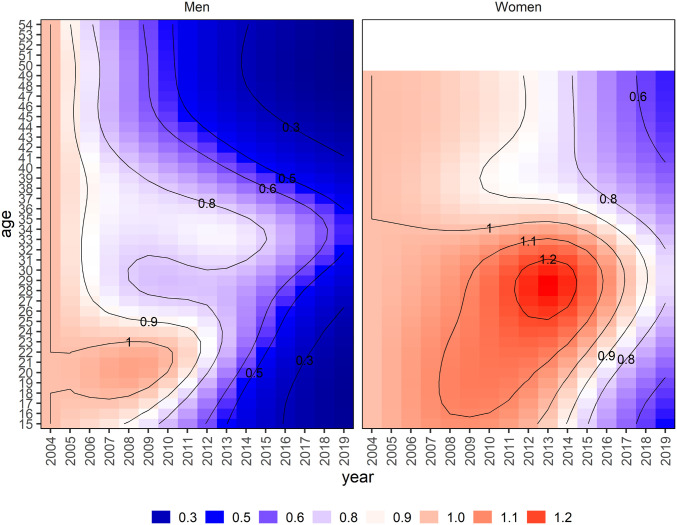
Smoothed age- and year-specific relative risks comparing age-specific incidence by year to age-specific incidence in 2004 (reference year).

The age distribution of HIV incidence in men and women remained stable throughout the earlier half of the study period (2004 to 2012) and shifted older in the latter half of follow-up (2012 to 2019) (Fig. 2 and see *SI Appendix* for all year-specific age distributions of incidence). HIV incidence declined (relative to 2004) first in the youngest men and women, with some evidence of early declines also observed in the oldest age-groups (Fig. 3). Incidence in women 25 to 34 y of age stayed relatively constant (with some indication of increase between 2008 to 2016) and declined the least of any age/sex subgroup (by <20% comparing 2019 levels to 2004 levels) (Fig. 3). Shifts in the incidence age distribution were the result of substantial declines in incidence (>50%) in men <age 30 and moderate declines in incidence (20 to 40%) in women <age 25.

**Fig. 4.**
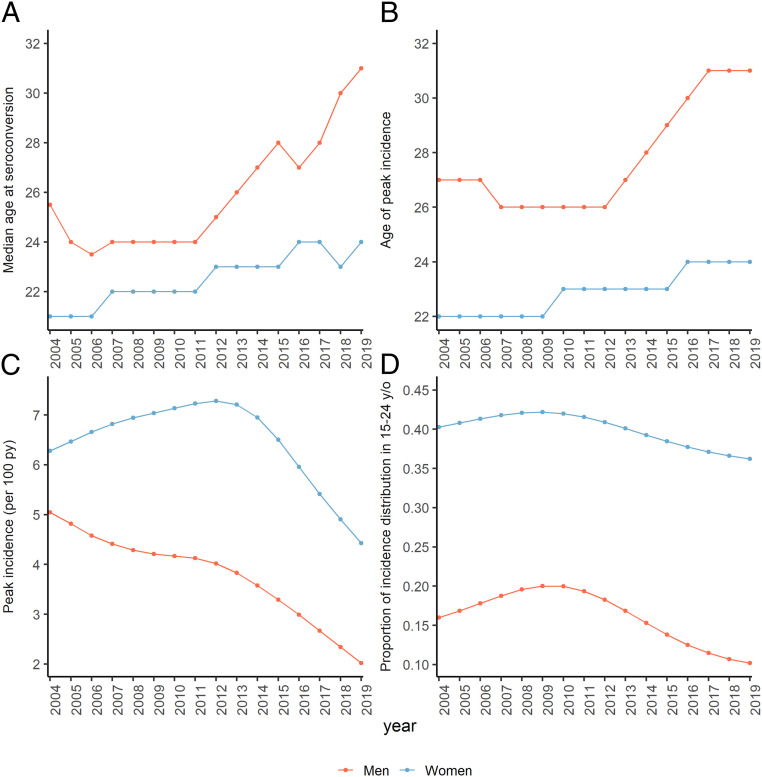
Annual sex-specific trends in (*A*) median age at seroconversion, (*B*) age of peak incidence, (*C*) peak age-specific HIV incidence (per 100 py), and (*D*) proportion of the incidence age distribution under age 25. (*B*–*D*) Derived from modeled age-specific HIV incidence; *A* is calculated as the median age at seroconversion across *n* = 300 imputed datasets.

The median age of seroconversion increased over the study period by 5.5 y in men (by 7.5 y from its low in 2006), from age 25.5 (IQR, 21.0 to 31.0) in 2004 to age 31.0 (IQR, 27.5 to 34.0) in 2019, and by 3.0 y in women, from its low at age 21.0 (IQR, 18.0 to 29.5) in 2004 to 24.0 (IQR, 20.0 to 27.0) in 2019 (Fig. 4*A*). The median age of the underlying HIV negative cohort increased by 3.0 y in men and 4.0 y in women. The age with the highest incidence (i.e., age of peak incidence) shifted older by 5 y in men between 2012 to 2019 (from age 26 to 31 y old) with no substantial change prior to 2012 (Fig. 4*B*). In women, the age of peak incidence increased by 2 y over the study period (age 22 y in 2004, 23 y in 2012, and 24 y in 2019). The highest age-specific incidence in men declined steadily from 5.0 per 100 py in 2004 to 2.0 per 100 py in 2019, with the steepest declines occurring after 2012. In women, the highest age-specific incidence initially rose from 6.3 per 100 py in 2004 to 7.3 per 100 py in 2012 after which it declined to its lowest level of 4.4 per 100 py in 2019 (Fig. 4*C*). The proportion of the incidence age distribution <age 25 (calculated as the area under the age-incidence curve) declined in men from its max of 20.0% in 2009 to 10.2% in 2019 and declined in women from a max of 42.2% in 2009 to 36.2% in 2019 (Fig. 4*D*).

**Fig. 5.**
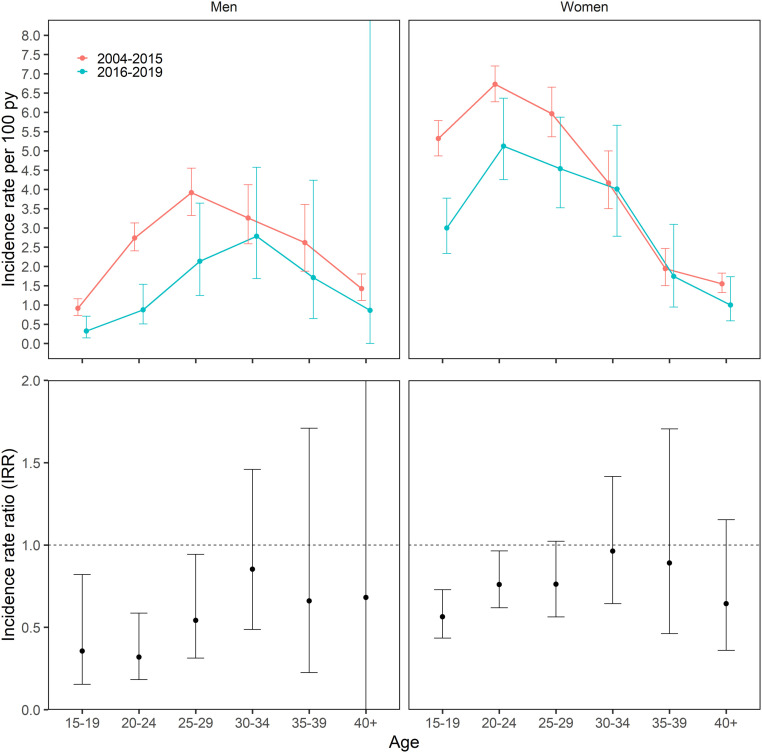
(*Top*) Fitted age distribution of HIV incidence for pre-UTT (2004 to 2015) and post-UTT (2016 to 2019). (*Bottom*) Categorical, age-specific IRRs comparing incidence post-UTT (2016 to 2019) to incidence pre-UTT (2004 to 2015).

In analyses using categorical age (5-y bins) comparing the periods before and after UTT went into effect in 2016, the age of peak incidence in men shifted from age 25 to 29 y, incidence rate (IR) = 3.9, 95% CI (3.3 to 4.6) to age 30 to 34 y, IR = 2.8, 95% CI (1.7 to 4.6). The peak incidence in women remained constant at 20 to 24 y in both the pre-UTT, IR = 6.7, 95% CI (6.3 to 7.2) and post-UTT eras, IR = 5.1, 95% CI (4.3 to 6.4) (Fig. 5, numerical values in Table 2). Incidence declined to the largest extent in young men and women comparing the periods before and after UTT went into effect in 2016. Incidence declined by 64.4% (IRR = 0.36, 95% CI, 0.15 to 0.82) in men aged 15 to 19 y, 68.1% (IRR = 0.32, 95% CI, 0.18 to 0.59) in men aged 20 to 24 y, and 45.8% (IRR = 0.54, 95% CI, 0.31 to 0.94) in men 25 to 29 and declined by 43.5% (IRR = 0.56, 95% CI, 0.43 to 0.73) in women aged 15 to 19 y, 24.0% (IRR = 0.76, 95% CI, 0.62 to 0.96) in women aged 20 to 24 y, and 24.0% (IRR = 0.76, 95% CI, 0.56 to 1.02) in women aged 25 to 29 y. (Fig. 5, numerical values in Table 2). In models adjusted for male circumcision status, incidence similarly declined to the largest extent in young men; there was a 56.8% decline (IRR = 0.43, 95% CI, 0.21 to 0.95) in men 15 to 19 y of age and a 61.6% decline (IRR = 0.38, 95% CI, 0.22 to 0.67) in men 20 to 24 y of age, with no significant evidence of decline in men >age 25 (Table 2). Age-specific incidence rates comparing pre- to post-UTT were also similar in models including inverse probability survey weights to account for potential selection bias attributed to differential probabilities of repeat testing by age, migration status, and community-level HIV prevalence (*SI Appendix*).

**Table 2.** Age-, sex-, and male circumcision status–specific incidence rates per 100 py and IRRs comparing the periods before (2004 to 2015) and after (2016 to 2019) UTT went into effect

Sex	Age-group	Incidence rate (per 100 py)		IRR
		2004 to 2015	2016 to 2019	
Women	15 to 19	5.32 (4.87 to 5.79)	3.00 (2.33 to 3.77)	**0.56 (0.43 to 0.73)**
	20 to 24	6.73 (6.27 to 7.20)	5.12 (4.25 to 6.37)	**0.76 (0.62 to 0.96)**
	25 to 29	5.96 (5.37 to 6.65)	4.53 (3.52 to 5.88)	0.76 (0.56 to 1.02)
	30 to 34	4.17 (3.50 to 4.99)	4.01 (2.78 to 5.66)	0.96 (0.64 to 1.42)
	35 to 39	1.94 (1.50 to 2.47)	1.74 (0.95 to 3.09)	0.89 (0.46 to 1.71)
	40 to 49	1.55 (1.32 to 1.83)	1.00 (0.59 to 1.73)	0.64 (0.36 to 1.15)
Men	15 to 19	0.92 (0.72 to 1.16)	0.32 (0.14 to 0.71)	**0.36 (0.15 to 0.82)**
	20 to 24	2.74 (2.40 to 3.13)	0.87 (0.51 to 1.53)	**0.32 (0.18 to 0.59)**
	25 to 29	3.91 (3.32 to 4.55)	2.13 (1.24 to 3.64)	**0.54 (0.31 to 0.94)**
	30 to 34	3.26 (2.59 to 4.12)	2.78 (1.68 to 4.57)	0.85 (0.49 to 1.46)
	35 to 39	2.62 (1.87 to 3.60)	1.71 (0.65 to 4.24)	0.66 (0.22 to 1.71)
	40 to 54	1.43 (1.12 to 1.80)	0.86 (0.00-Inf)	0.68 (0.00-Inf)
Men (circumcised)*	15 to 19	0.52 (0.35 to 0.76)	0.22 (0.11 to 0.48)	**0.43 (0.21 to 0.95)**
	20 to 24	1.58 (1.12 to 2.24)	0.60 (0.33 to 1.06)	**0.38 (0.22 to 0.67)**
	25 to 29	2.24 (1.52 to 3.24)	1.38 (0.76 to 2.41)	0.62 (0.36 to 1.09)
	30 to 34	1.89 (1.26 to 2.84)	1.74 (0.99 to 3.08)	0.92 (0.54 to 1.56)
	35 to 39	1.50 (0.94 to 2.37)	1.11 (0.41 to 2.94)	0.75 (0.27 to 2.06)
	40 to 54	0.82 (0.53 to 1.20)	0.18 (0.00-Inf)	0.21 (0.00-Inf)
Men (uncircumcised)*	15 to 19	0.93 (0.74 to 1.18)	0.40 (0.19 to 0.84)	
	20 to 24	2.82 (2.48 to 3.21)	1.08 (0.63 to 1.84)	
	25 to 29	4.02 (3.38 to 4.69)	2.46 (1.49 to 4.20)	
	30 to 34	3.37 (2.64 to 4.32)	3.06 (1.99 to 5.04)	
	35 to 39	2.68 (1.96 to 3.66)	1.98 (0.78 to 5.20)	
	40 to 54	1.44 (1.13 to 1.84)	0.30 (0.00-Inf)	

IRR = incidence rate ratio comparing age-specific incidence in the UTT era (2016 to 2019) relative to age-specific incidence in the pre-UTT era (2004 to 2015). Bold IRRs indicate significance at alpha < 0.05.

* Incidence rates for circumcised and uncircumcised men are estimated by fitting a generalized linear model with an indicator for individual-level circumcision status (circumcision model). Fitted IRR’s from the circumcision model are adjusted for circumcision status without an interaction term and so are equivalent for both uncircumcised and circumcised men.

## Discussion

We observed a marked age shift in the distribution of HIV-1 incidence between 2004 and 2019 in one of the world’s largest population-based HIV cohorts in rural KwaZulu-Natal, South Africa. HIV incidence shifted to older age-groups because incidence declines were large among both young men (<age 30) and young women (<age 25) and small among older adults. Age shifts in male incidence occurred earlier and were larger than those in women. This finding likely reflects a delay in the indirect protection afforded to the female partners of lower-incidence young men as they age (52, 53). Our results provide direct epidemiological evidence of the changing demographics of HIV risk in SSA in the era of large-scale HIV treatment and prevention and highlight the need to re-evaluate age targets for intervention strategies going forward.

Several HIV-specific epidemic dynamics may explain the observed age shifts in HIV incidence. First, age-specific targeting of primary prevention, including the scale-up of VMMC over the past decade, has likely played a role in reducing HIV risk disproportionately in younger male cohorts and, to some extent, in the young female partners of those men as they age. In our analysis, the prevalence of male circumcision increased to the largest extent among young men (though men >25 y of age also experienced moderate increases in coverage). However, when adjusting models for circumcision status, we observed a similar pattern as in unadjusted models: larger incidence declines in young men (<25) compared to older men, indicating that differential VMMC targeting and uptake in younger men is not the primary factor driving age shifts in risk.

Second, uneven changes in age-specific population viremia over time may have shifted the age distribution of risk older. HIV incidence is inextricably linked to the population prevalence of detectable viral load, a population-level biomarker that combines the prevalence of HIV and detectable viral load among PLHIV to estimate the background risk of HIV acquisition (32, 54). While older age-groups tend to have the highest ART coverage (2, 3, 55), the prevalence of HIV infection in SSA has more than doubled in those >age 25 in the past two decades (13, 14, 21, 29), a result of both the natural aging of high incidence cohorts and the increase in life expectancy of PLHIV on suppressive ART (17–19, 56, 57). The counteracting effects of decreasing viral load among PLHIV and increasing HIV prevalence of aging cohorts results in persistently elevated prevalence of detectable viremia in some older age-groups (5, 29, 58, 59). In our study, despite the expanding coverage of ART and VMMC, men and women in the 25- to 34-y age-group experienced minimal changes in incidence over time. Stable incidence in this group may reflect constant population prevalence of detectable viral load in the corresponding age-groups of their partners. In contrast, younger cohorts benefit from both the direct effects of increasing ART coverage and the indirect effects of immediate reductions in prevalence from declining incidence. Thus, even modest increases in ART coverage can produce disproportionately larger declines in incidence in younger age-groups. Additionally, though not significant, we did observe some evidence of declines in incidence in the oldest women (40 to 49 y of age) and men (40 to 54 y of age), which may reflect larger increases in ART coverage in the oldest age-groups compared to those 25 to 39 y of age. Further study can confirm this hypothesis by testing for differential changes in population viremia over time by age and sex.

Third, declines in the overall HIV transmission rate may further delay the time to infection, shifting the distribution of new infections to older. The inverse relationship between the force of infection and age at infection has been observed across infectious disease systems in which early exposure and immunity provide some degree of subsequent protection (35, 36). For life-long diseases like HIV, a reduced force of infection can shift the distribution of risk older by changing the risk profile of population cohorts as they age. Under a lower force of infection, individuals with greater biological and behavioral susceptibility to HIV remain in the population at risk for a longer time (60), thereby increasing the susceptibility profile of the older population at risk compared to earlier birth cohorts. Additional study is needed to test this hypothesis over longer time periods. Dynamic changes in the age distribution of risk must further be evaluated against demographic shifts in SSA’s age structure, which is expected to become younger over time. Thus, even as overall HIV risk declines, the absolute number of new infections among youth may increase (61).

Age shifts in HIV incidence in high-burden communities have major implications for realigning demographic targets for prevention to include older age-groups. While the continued focus of HIV prevention on younger populations, especially adolescent girls and young women, remains a crucial component of population-level risk reduction (62, 63), more attention is needed to address lagging incidence declines among older individuals. Expanding age targets for ongoing prevention programs (including pre-exposure prophylaxis and VMMC) may be necessary to address a changing demographic landscape of risk in SSA over the coming decade. HIV prevention methods with limited effectiveness in younger age-groups may prove effective in older age-groups because of better uptake, adherence, and product efficacy (64, 65) and could be reconsidered as effective preventive modalities with expanded age targeting.

Our study’s strengths include the use of one of the world’s largest population-based HIV cohort to estimate incidence—the gold standard approach to directly measure changes in population-level incidence—over a 16-y period spanning the scale-up of ART and VMMC. We employed flexible models to capture nonlinear trends in age- and sex-specific incidence and quantify changes in the age distribution of risk over time. Nonresponse or refusal to participate in annual HIV testing is common in the AHRI surveillance area (ranging from 34 to 60%; *SI Appendix*) (13, 46), similar to that in other regions in South Africa (66). We conducted further sensitivity analysis to ensure our results were robust to differences in testing participation over time. We found no differences in age- and gender-specific incidence rates (comparing pre- to post-UTT era) in analyses weighted for factors associated with HIV testing probability that may have biased the primary results. We further incorporated uncertainty in seroconversion date (between last negative and first positive for those who seroconverted) into our estimates using the random-point imputation method for interval censored data, which has been shown to be robust to bias when data contain long censoring intervals and suboptimal testing rates (49).

The limitations of our study include the inability to account for differences in unmeasured behavioral factors over time that could account for some of the age-specific incidence trends. Disproportionate changes in sexual risk behavior in younger compared to older age-groups may account for some of the observed age shifts in risk. However, some indicators suggest that sexual risk behavior has not changed substantially over the course of the study period. Self-reported condom use has remained relatively stable in the cohort since 2012 (2), consistent with a lack of change in condom use in a nearby cohort (67). Further study is needed to quantify the relative contributions of changes in age-specific sexual behaviors (including changes in age of sexual debut, frequency of partnership turnover, and age of sexual partners) in addition to the effects of biomedical interventions.

Aging cohorts are expected to comprise an increasingly larger proportion of the population living with HIV across SSA in the UTT era (68–71), prompting research and programmatic efforts to address the public health challenges of an aging population living with HIV (57, 72–74). Strategies specifically tailored to older adults, and especially to older women (75), may be needed to bring incidence below epidemic control levels.

In conclusion, the age distribution of HIV incidence has shifted older in a high-burden community of South Africa, contemporaneous with the large-scale expansion of HIV treatment and prevention over the last decade. We hypothesize that age-specific disparities in treatment coverage and primary HIV prevention targeting, increasing prevalence of HIV in older age-groups, and delayed age at infection contribute to age shifts in HIV risk. The changing demographics of the HIV epidemic in high-burden communities have implications for the design of interventions and may require expanding the scope of HIV prevention to include older age-groups (those >25) in the era of UTT, who will likely comprise a larger proportion of incidence as the HIV epidemic continues to contract.

## Supplementary Material

Supplementary File

## Data Availability

The datasets used in this study have been deposited in the AHRI data repository (https://data.ahri.org/index.php/home). To access the licensed datasets, the applicant must agree to the terms and conditions of use by completing an Application for Access to a Licensed Dataset. This request will be reviewed by the AHRI Data Release Committee, who may decide to approve the request, to deny access to the data, or to request additional information from the applicant.
