# Supplementary material for: Large age shifts in HIV-1 incidence patterns in KwaZulu-Natal, South Africa
Source: Proc Natl Acad Sci U S A. 2021 Jul 9;118(28):e2013164118. doi: 10.1073/pnas.2013164118 (PMC8285891; doi:10.1073/pnas.2013164118)
Supplement: Supplementary File [file pnas.2013164118.sapp.pdf]

PNAS
www.pnas.org

Supplementary Information for

Large age-shifts in HIV-1 incidence patterns in KwaZulu-Natal, South Africa

Adam Akullian, Alain Vandormael, Joel C. Miller, Anna Bershteyn, Edward Wenger, Diego Cuadros, Dickman Gareta, Till Bärnighausen, Kobus Herbst, and Frank Tanser

Corresponding author: Adam Akullian
Email: aakullian@idmod.org

This PDF file includes:

Supplementary text
Figures S1 to S5
Tables S1 to S2
SI References

Supplementary Information Text

Statistical analysis

Smoothed person-time incidence rates and 95% confidence intervals (CI) were estimated for men and women separately using Poisson generalized additive models (GAMs) with penalized, thin plate regression splines (1) (isotropic, locally weighted regression smoothers) and an offset for person-time at risk (2). The expected number of seroconversions $E(y_i)$ is the product of the Poisson distributed seroconversion rate λ_i and person time at risk T_i , and was modelled as a linear combination of 1- and 2-dimensional smooth terms:

$$\log[E(\mathrm{y}_i)] = \lambda_i T_i = \beta_0 + f_1(\mathrm{age}_i) + f_2(\mathrm{year}_i) + f_3(\mathrm{age}_i, \mathrm{year}_i) + offset[\log(\mathrm{T}_i)] + \varepsilon_i,$$

$$y_i \sim Poisson(\lambda_i),$$

where β_0 is an intercept, ε_i are identically distributed and independent (i.i.d.) errors, and $f_{1,\ldots,}f_p$ are smooth regression splines:

$$f(x) = \sum_{j=1}^{J} b_j(x)\, Z_j,$$

defined as the sum of piece-wise polynomial basis functions b_j and regression coefficients Z_j with basis dimension J

Circumcision-adjusted models

We estimated the distribution of male circumcision (MC) coverage (by self-report) by age and over time (Figure S3). Circumcision coverage was <5% across all age-groups before 2009 and increased to a larger extent among men <25 (~40% among men 20 years of age in 2016 compared to ~20% among men over 30). Most of the MC scale-up and resulting age-differences in MC coverage appeared later in the study period (after 2015) and thus the effects of MC scale-up on incidence may not show-up in directly observed incidence until more person-time can accrue.

In models adjusting for circumcision status (Figure S4), differential changes in MC coverage over time by age did not appear to be a large component age-shifts in incidence. Incidence distributions observed among men overall looked very similar to those of uncircumcised men because most of the person time is concentrated in uncircumcised men. In our models adjusting for individual-level circumcision status in men, the age-specific relative risks comparing incidence pre to post UTT were attenuated towards the null only slightly for most age-groups, suggesting that VMMC scale-up is playing only a minor role in incidence declines observed to-date and does not appear to be differential across age-groups. We observed similar shifts in incidence (large declines in young men, small declines in older men) when restricting our analysis to uncircumcised men or circumcised men (see Figure 5 below).

Fig. S1.

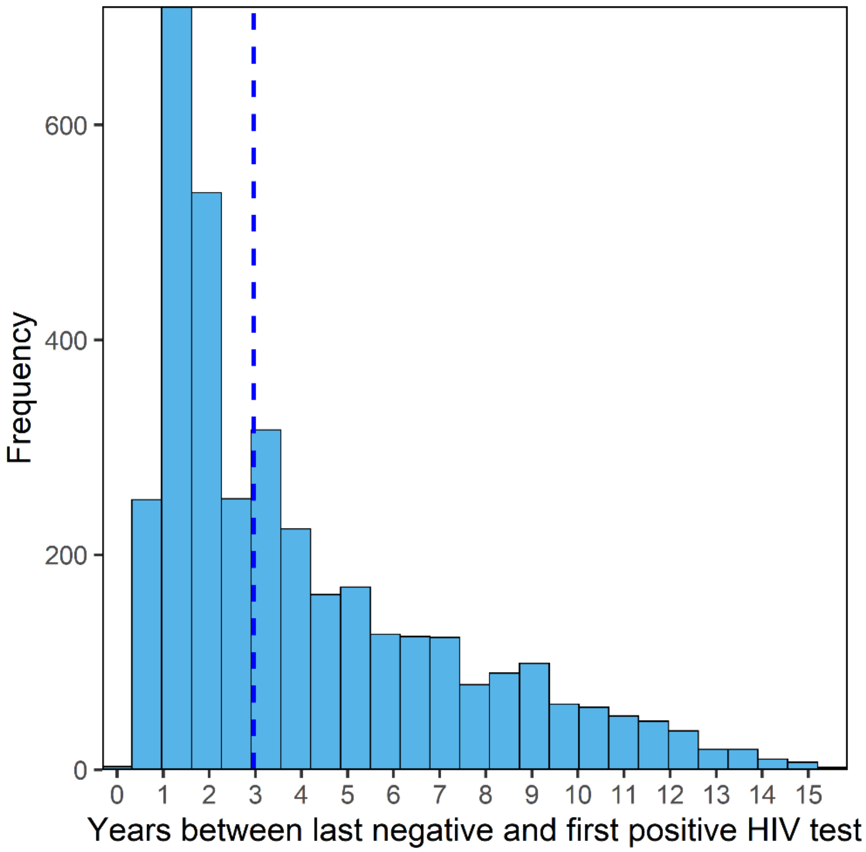

Distribution of time between last negative and first positive (censoring interval) for individuals in the incidence cohort who seroconverted. Dashed blue line indicates median censoring interval of 3 years (IQR = 1.4-5.8 years)

Fig. S2.

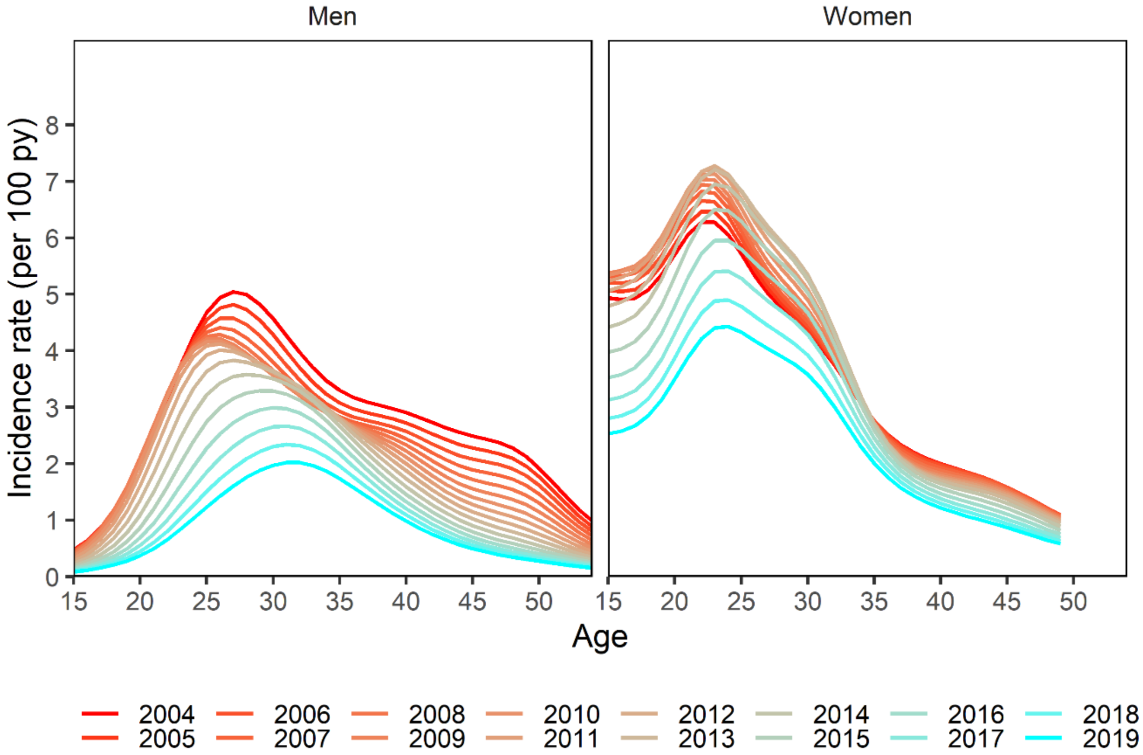

Smoothed, sex-specific age-distribution of HIV incidence by year (2004-2019) from the fitted generalized additive model.

Fig. S3.

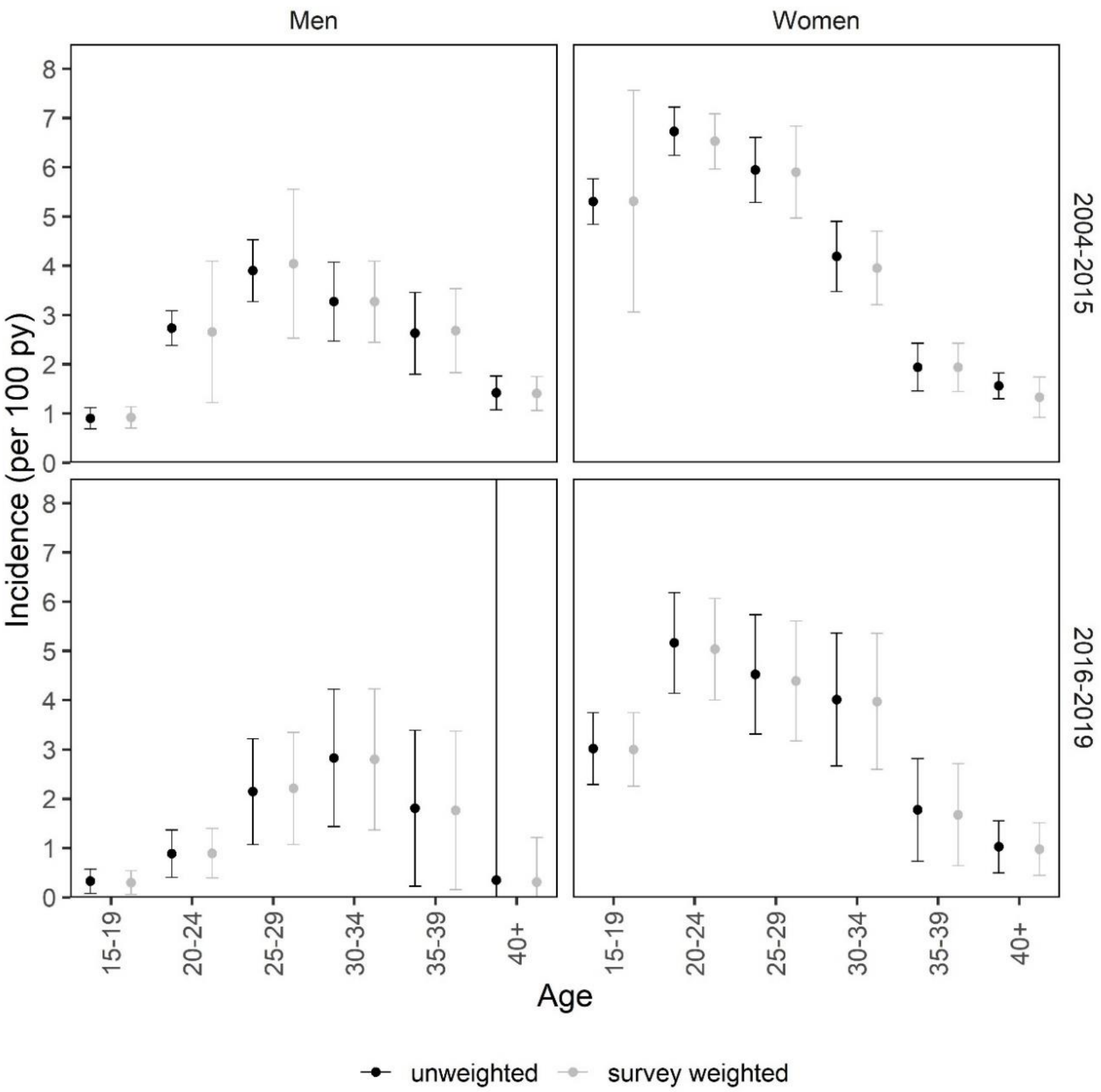

Comparison of age- and period-specific incidence estimates from categorical models fit without survey weights (unweighted) and with survey weights (svy). Individual survey weights were estimated by year using a logistic regression model with missingness regressed on continuous age, number of out-migrations and duration of out-migrations, and HIV prevalence in the surrounding community.

Figure S4

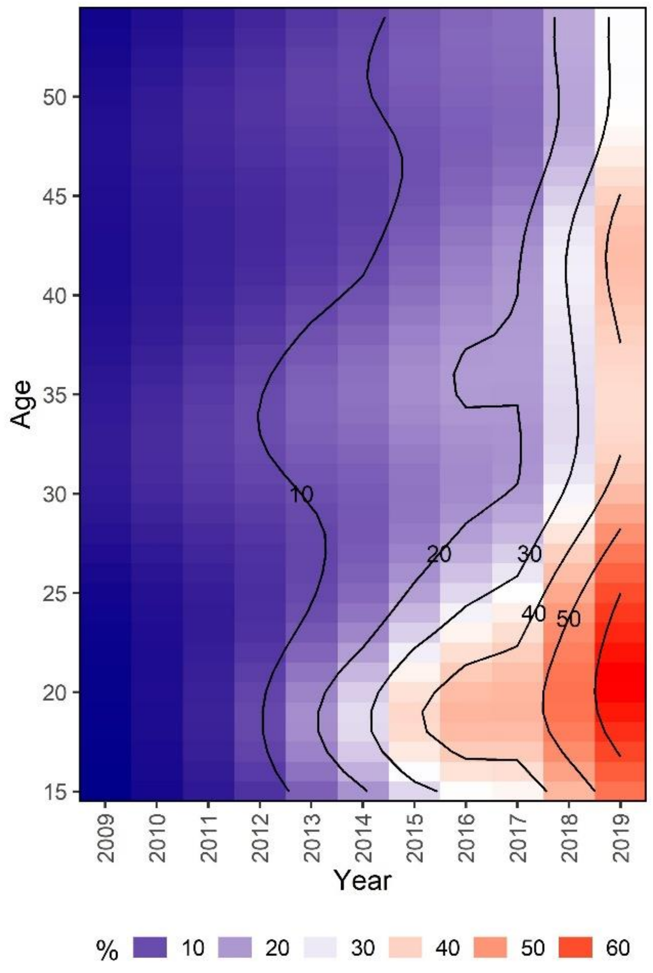

Male circumcision prevalence by continuous age and year between 2009 and 2019 from a fitted generalized additive logistic regression model with a smooth interaction between age and year

Figure S5

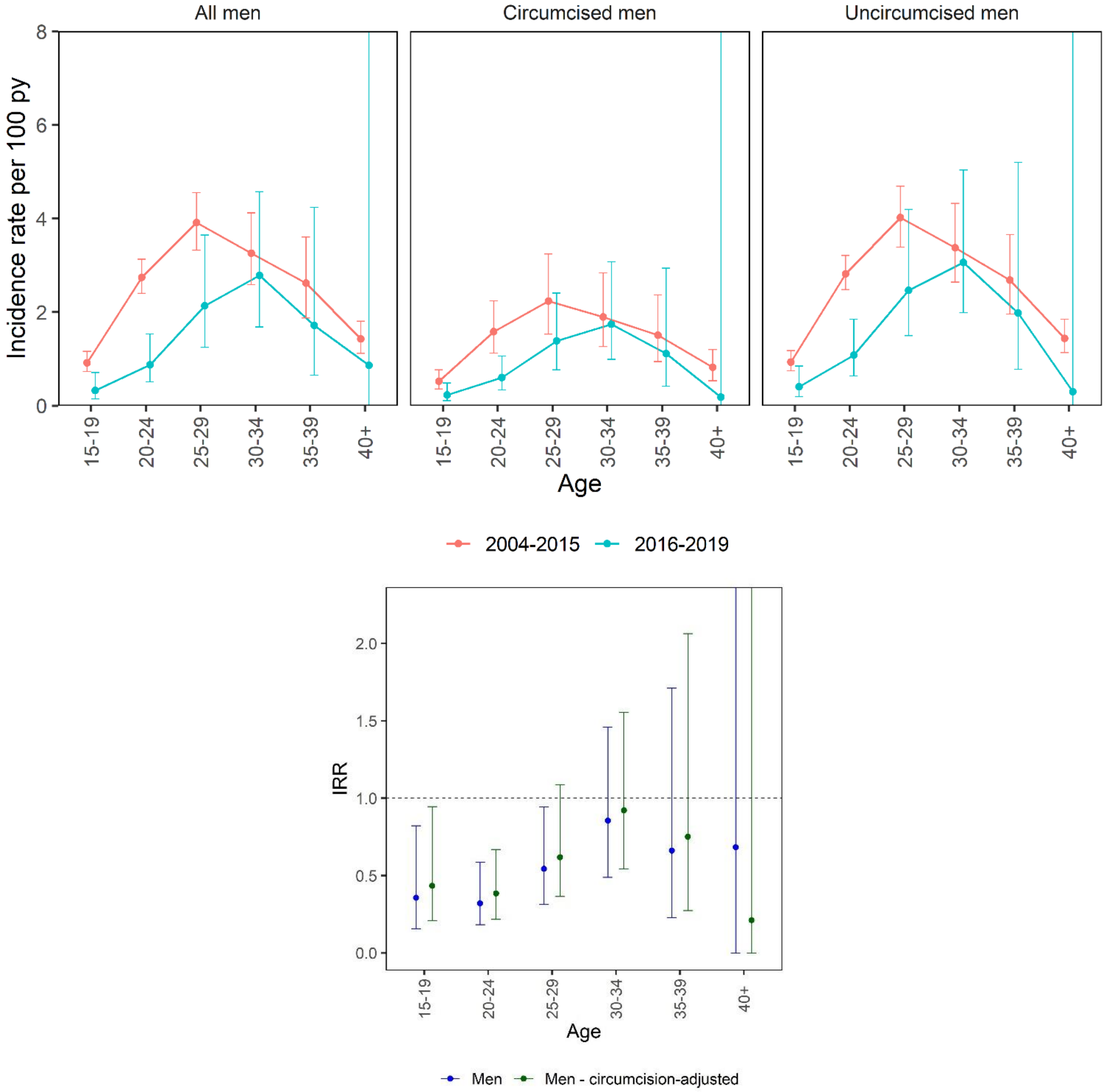

(Top panel) Male incidence in the periods before (2004-2015) versus after (2016-2019) universal test and treat (UTT) went into by self-reported circumcision status. (Bottom panel) Incidence rate ratios comparing incidence pre-versus post UTT in unadjusted models (blue) and in models adjusted for circumcision status (green).

Table S1. Summary of participation in HIV testing and inclusion in the longitudinal HIV incidence cohort by year.

	**Annual HIV testing**						**HIV incidence cohort (cumulative)**			
Year	Eligible[a]/ enumerated[b]	(%)	Contacted[c]/ eligible	(%)	Tested[d] / contacted	(%)	Ever tested[e]	HIV negative[f]	Repeat test[g]	%
2004	15,748/15,803	(99.7)	15,748/15,748	(100)	9,402/15,748	(59.7)	57.2	9,957	7,370	(74.0)
2005	25,203/26,477	(95.2)	22,105/25,203	(87.7)	9,422/22,105	(42.6)	54.4	13,488	9,937	(73.7)
2006	23,536/26,209	(89.8)	21,538/23,536	(91.5)	8,643/21,538	(40.1)	58.7	15,767	11,512	(73.0)
2007	28,364/32,844	(86.4)	25,689/28,364	(90.6)	9,957/25,689	(38.8)	58.4	17,610	12,805	(72.7)
2008	31,020/36,228	(85.6)	28,859/31,020	(93.0)	10,032/28,859	(34.8)	59.8	19,371	14,041	(72.5)
2009	27,077/32,072	(84.4)	24,795/27,077	(91.6)	8,858/24,795	(35.7)	64.9	20,381	14,669	(72.0)
2010	32,173/34,968	(92.0)	26,520/32,173	(82.4)	11,228/26,520	(42.3)	66.8	22,674	16,176	(71.3)
2011	32,127/32,978	(97.4)	25,586/32,127	(79.6)	10,385/25,586	(40.6)	69.7	24,167	17,029	(70.5)
2012	30,506/32,327	(94.4)	23,145/30,506	(75.9)	7,916/23,145	(34.2)	68.7	25,604	17,974	(70.2)
2013	30,768/32,964	(93.3)	24,840/30,768	(80.7)	9,912/24,840	(39.9)	71.8	27,545	19,247	(69.9)
2014	31,225/33,013	(94.6)	24,471/31,225	(78.4)	9,541/24,471	(39.0)	73.4	29,032	20,291	(69.9)
2015	30,509/32,211	(94.7)	27,085/30,509	(88.8)	13,096/27,085	(48.4)	77.4	31,087	21,586	(69.4)
2016	32,502/34,044	(95.5)	28,239/32,502	(86.9)	14,737/28,239	(52.2)	79.8	33,370	22,733	(68.1)
2017	31,687/34,542	(91.7)	26,240/31,687	(82.8)	11,430/26,240	(43.6)	80.1	34,698	23,281	(67.1)
2018	32,052/33,223	(96.5)	23,990/32,052	(74.8)	12,641/23,990	(52.7)	82.1	36,409	23,281	(63.9)
Average		(92.7)		(85.7)		(43.0)				(70.9)

Data on eligibility unavailable for 2019

a. Eligibility defined as ≥ 15 years of age, mentally able, and resident of the household.
b. All individuals identified by a key household informant as residing in the household.
c. Present in the household at time of annual HIV testing survey
d. Provided dried blood spot for HIV testing
e. Percent having ever tested for HIV among those eligible and contacted since annual testing began in 2003
f. Cumulative number individuals ever having tested HIV negative and 15-49 (women) or 15-54 (male) and thus eligible for follow-up in the HIV incidence cohort
g. Cumulative number of 15-49 (women) or 15-54 (male) HIV negative individuals and having received a repeat test

Table S2. Demographic and behavioral characteristics of the entire sample of individuals who tested for HIV at least once between 2004-2019 by sex and testing group [One test negative, only positive tests, and repeat tester (HIV negative cohort)]. Data shown include "missing" category to compare probability of missing data by variable. Categorical variables with no "missing" category are complete.

	One negative test		Only positive tests		Repeat tester cohort	
Sex	Male	Female	Male	Female	Male	Female
N	7273<br>n (%) / mean (sd)	5755<br>n (%) / mean (sd)	3071<br>n (%) / mean (sd)	7464<br>n (%) / mean (sd)	9801<br>n (%) / mean (sd)	12605<br>n (%) / mean (sd)
Age (years)	24.60 (9.50)	25.05 (10.08)	35.09 (9.41)	32.33 (8.58)	25.67 (9.22)	26.91 (9.51)
15-19	2891 (39.7)	2426 (42.2)	213 (6.9)	566 (7.6)	2823 (28.8)	3318 (26.3)
20-24	2001 (27.5)	1396 (24.3)	238 (7.7)	1111 (14.9)	3492 (35.6)	3973 (31.5)
25-29	973 (13.4)	607 (10.5)	463 (15.1)	1424 (19.1)	1455 (14.8)	1811 (14.4)
30-34	447 (6.1)	312 (5.4)	629 (20.5)	1471 (19.7)	581 (5.9)	796 (6.3)
35-39	248 (3.4)	193 (3.4)	557 (18.1)	1219 (16.3)	359 (3.7)	670 (5.3)
40-54	713 (9.8)	821 (14.3)	971 (31.6)	1673 (22.4)	1091 (11.1)	2037 (16.2)
Ever migrated away from study area						
No	2354 (32.4)	2041 (35.5)	1404 (45.7)	3192 (42.8)	3588 (36.6)	5316 (42.2)
Yes	4919 (67.6)	3714 (64.5)	1667 (54.3)	4272 (57.2)	6213 (63.4)	7289 (57.8)
Ever experienced unemployment*						
No	1371 (22.6)	925 (19.8)	980 (33.7)	1384 (19.0)	1052 (11.7)	913 (7.8)
Yes	4684 (77.4)	3753 (80.2)	1927 (66.3)	5886 (81.0)	7974 (88.3)	10776 (92.2)
Missing	1189 (16.4)	1186 (20.2)	83 (2.8)	160 (2.2)	484 (5.1)	632 (5.1)
ART initiation (among HIV+)						
No clinical record			1974 (64.3)	4105 (55.0)	672 (75.8)	1716 (63.9)
Initiated ART			1097 (35.7)	3359 (45.0)	215 (24.2)	971 (36.1)
Circumcised (among men)						
No	2883 (65.9)		1637 (80.3)		5147 (62.9)	
Yes	1494 (34.1)		401 (19.7)		3039 (37.1)	

Missing	2896 (39.8)		1033 (33.6)		1615 (16.5)	
Age first married	30.99 (6.58)	25.80 (6.12)	31.94 (6.67)	26.48 (6.41)	30.60 (6.48)	25.43 (6.37)
Ever had sex						
No	2037 (54.4)	1939 (42.9)	167 (11.4)	248 (3.9)	2177 (29.8)	2287 (18.7)
Yes	1705 (45.6)	2579 (57.1)	1300 (88.6)	6069 (96.1)	5127 (70.2)	9965 (81.3)
Missing	3531 (48.5)	1237 (21.5)	1604 (52.2)	1147 (15.4)	2497 (25.5)	353 (2.8)
Age at first sex (years)	17.05 (2.57)	17.89 (2.50)	17.80 (3.01)	17.49 (2.30)	16.84 (2.50)	17.47 (2.25)
Lifetime partners (number)	3.40 (4.13)	1.72 (1.26)	5.14 (6.19)	2.70 (2.48)	3.80 (4.52)	2.09 (1.97)

Percentages calculated out all non-missing values. “Missing” category refers to refusals, not applicable, or not present at the time of survey and is calculated out of the total sample size in the strata.
*Unemployment information does not include data from 2019 as the question was no longer asked.

SI References

1. S. Wood, *Generalized additive models: an introduction with R* (CRC press, 2006).
2. T. Webster, V. Vieira, J. Weinberg, A. Aschengrau, Method for mapping population-based case-control studies: an application using generalized additive models. *Int J Health Geogr* **5**, 26 (2006).
